# Supplementary material for: Mechanisms governing the pioneering and redistribution capabilities of the non-classical pioneer PU.1
Source: Nat Commun. 2020 Jan 21;11:402. doi: 10.1038/s41467-019-13960-2 (PMC6972792; doi:10.1038/s41467-019-13960-2)
Supplement: Supplementary file 7 — Source data [file 41467_2019_13960_MOESM7_ESM.zip › Source_Data/FigureS2/FigureS2B_MotifScanOutput/KmeansCluster2/homerResults/motif20.similar.html]

motif20

## Information for motif20

C
A
T
G
C
T
G
A
T
C
G
A
C
T
G
A
A
G
T
C
C
A
G
T
A
C
G
T
C
T
G
A
G
A
T
C
T
C
G
A
C
T
G
A
A
C
G
T
T
A
G
C
  
Reverse Opposite:  

A
C
T
G
T
C
G
A
G
A
C
T
A
C
G
T
C
T
A
G
G
A
C
T
T
C
G
A
G
T
C
A
C
T
A
G
A
G
C
T
A
C
G
T
A
G
C
T
G
A
T
C
  

|  |  |
| --- | --- |
| p-value: | 1e-55 |
| log p-value: | -1.268e+02 |
| Information Content per bp: | 1.885 |
| Number of Target Sequences with motif | 115.0 |
| Percentage of Target Sequences with motif | 2.29% |
| Number of Background Sequences with motif | 91.4 |
| Percentage of Background Sequences with motif | 0.22% |
| Average Position of motif in Targets | 93.5 +/- 73.2bp |
| Average Position of motif in Background | 100.8 +/- 68.3bp |
| Strand Bias (log2 ratio + to - strand density) | 0.4 |
| Multiplicity (# of sites on avg that occur together) | 1.00 |
| Motif File: | file (matrix) reverse opposite |

### Similar de novo motifs found

|  |  |  |  |  |  |  |  |
| --- | --- | --- | --- | --- | --- | --- | --- |
| Rank | Match Score | Redundant Motif | P-value | log P-value | % of Targets | % of Background | Motif file |
| 1 | 0.974 | C A T G C G T A T C G A C T G A A G T C A C G T A C G T C G T A G A T C T G C A C T G A A C G T A G T C C T G A | 1e-53 | -123.635847 | 2.43% | 0.28% | motif file (matrix) |
| 2 | 0.959 | C A T G C T G A T C G A G C T A A G T C C A G T A G C T C T G A G A T C T C G A C T G A A G C T | 1e-51 | -118.261905 | 2.43% | 0.30% | motif file (matrix) |
| 3 | 0.914 | C T G A G C T A A G T C C A G T A C G T C T G A G A T C T C G A C T G A A C G T G A T C | 1e-43 | -100.141924 | 2.19% | 0.30% | motif file (matrix) |
| 4 | 0.929 | C A T G C T G A C T G A C G T A G T A C G C A T A G C T C T G A A G T C C G T A C G T A A G C T A G T C C T G A | 1e-34 | -79.489229 | 1.57% | 0.19% | motif file (matrix) |
| 5 | 0.845 | T G C A C T G A A G T C G A C T C A G T C T G A G T A C G C T A C T G A C A G T A G T C C G T A | 1e-33 | -77.070217 | 3.28% | 0.95% | motif file (matrix) |
